# Two-Component System RgfA/C Activates the *fbsB* Gene Encoding Major Fibrinogen-Binding Protein in Highly Virulent CC17 Clone Group B Streptococcus

**DOI:** 10.1371/journal.pone.0014658

**Published:** 2011-02-04

**Authors:** Rim Al Safadi, Laurent Mereghetti, Mazen Salloum, Marie-Frédérique Lartigue, Isabelle Virlogeux-Payant, Roland Quentin, Agnès Rosenau

**Affiliations:** 1 Equipe d'Accueil 3854 Bactéries et risque materno-fœtal, Institut Fédératif de Recherche 136 Agents Transmissibles et Infectiologie, UFR Médecine, Université François Rabelais de Tours, Tours, France; 2 Institut National de la Recherche Agronomique UR1282 Infectiologie Animale et Santé Publique, Nouzilly, France; 3 Service de Bactériologie-Virologie, Hôpital Bretonneau, CHRU de Tours, Tours, France; 4 Service de Bactériologie et Hygiène Hospitalière, Hôpital Trousseau, CHRU de Tours, Tours, France; Columbia University, United States of America

## Abstract

Group B streptococcus (GBS) strains with the highest ability to bind to human fibrinogen belong to the highly invasive clonal complex (CC) 17. To investigate the fibrinogen-binding mechanisms of CC17 strains, we determined the prevalence of fibrinogen-binding genes (*fbsA* and *fbsB*), and *fbs* regulator genes (*rogB* encoding an *fbsA* activator, *rovS* encoding an *fbsA* repressor and *rgf* encoding a two-component system [TCS] whose role on *fbs* genes was not determined yet) in a collection of 134 strains representing the major CCs of the species. We showed that specific gene combinations were related to particular CCs; only CC17 strains contained the *fbsA*, *fbsB*, and *rgf* genes combination. Non polar *rgfAC* deletion mutants of three CC17 serotype III strains were constructed. They showed a 3.2- to 5.1-fold increase of *fbsA* transcripts, a 4.8- to 6.7-fold decrease of *fbsB* transcripts, and a 52% to 68% decreased fibrinogen-binding ability, demonstrating that the RgfA/RgfC TCS inhibits the *fbsA* gene and activates the *fbsB* gene. The relative contribution of the two *fbs* genes in fibrinogen-binding ability was determined by constructing isogenic *fbsA*, *fbsB*, deletion mutants of the three CC17 strains. The ability to bind to fibrinogen was reduced by 49% to 57% in *ΔfbsA* mutants, and by 78% to 80% in *ΔfbsB* mutants, suggesting that FbsB protein plays a greater role in the fibrinogen-binding ability of CC17 strains. Moreover, the relative transcription level of *fbsB* gene was 9.2- to 12.7-fold higher than that of *fbsA* gene for the three wild type strains. Fibrinogen-binding ability could be restored by plasmid-mediated expression of *rgfAC*, *fbsA*, and *fbsB* genes in the corresponding deletion mutants. Thus, our results demonstrate that a specific combination of *fbs* genes and *fbs* regulator genes account for the high fibrinogen-binding ability of CC17 strains that may participate to their enhanced invasiveness for neonates as compared to strains of other CCs.

## Introduction

*Streptococcus agalactiae* (group B streptococcus [GBS]) frequently asymptomatically colonizes the intestinal and/or urogenital tract of humans. It is the leading cause of invasive infections in neonates and has emerged as an increasingly cause of invasive diseases in immunocompromised and elderly adults [1], [2]. Several studies have emphasized the clonal structure of GBS species and demonstrated that GBS diseases are mostly caused by a limited set of clonal lineages [3]–[8]. Indeed, strains belonging to clonal complex (CC) 17 appear to be strongly able to invade the central nervous system (CNS) of neonates [9]–[13]. The remarkable homogeneity within this highly virulent lineage is likely of importance for disease pathogenesis, though few studies have been conducted to identify specific differences in virulence characteristics between lineages.

Various molecules either secreted or located at the bacterial surface account for the pathogenicity of GBS strains [14]–[17]. Among these molecules, FbsA and FbsB are proteins with no structural homology which both bind to human fibrinogen, mediate the bacterial adhesion to or invasion of epithelial and endothelial cells, and contribute to the bacterial escape from the immune system [18]–[22]. Deletion of the *fbsB* gene that has been described for a unique strain belonging to CC23 phylogenetic lineage, did not attenuate its fibrinogen-binding ability; conversely, deletion of the *fbsA* gene in this strain resulted in a loss of fibrinogen-binding activity, thus suggesting that FbsA protein was the major fibrinogen-binding protein in GBS [18], [20], [21]. However, while studying a collection of 111 human strains, we showed that the presence of the sole *fbsA* gene was not sufficient to result in strong binding ability to fibrinogen [17]. Indeed, the population of strains with the significantly highest ability to bind to fibrinogen had both the *fbsB* and *fbsA* genes and belonged to CC17 phylogenetic lineage [17]. Thus, the role of *fbs* genes and in particular the *fbsB* gene in the fibrinogen-binding ability of CC17 strains remains unclear and requires further investigation.

Two transcriptional regulators were shown to control the *fbsA* gene transcription in a CC23 GBS strain: RogB, a member of the RALP (RofA-like proteins) family, exerts a positive effect [23], and RovS, relative to the Rgg family of Gram-positive transcriptional regulators, exerts a negative effect [24]. In addition, Spellerberg et al. described a two-component system (TCS), the regulator of fibrinogen-binding *rgfBDAC* operon that principally encodes the response regulator RgfA and the histidine kinase RgfC [25]. Disruption of the *rgfC* gene altered the bacterial binding to fibrinogen. The role of *rgf* locus on *fbs* genes transcription was not studied yet, and it can be speculated that *fbsA* and/or *fbsB* genes are under the transcriptional control of the RgfA/RgfC TCS.

To investigate the mechanisms allowing the high fibrinogen-binding ability of CC17 strains (i) we determined the *fbs* genes and *fbs* regulator genes profile of 38 CC17 strains as compared to 96 GBS strains of the other four major phylogenetic lineages constituting this species, and found that specific gene combinations were related to particular CCs; (ii) we constructed non polar *rgfAC* deletion mutants of three CC17 strains in order to determine the role of *rgf* locus on *fbs* genes transcription; (iii) we quantified the transcription levels of *fbsA* and *fbsB* genes of these three strains, and constructed their *fbsA* and *fbsB* deletion mutants in order to determine the relative contribution of *fbs* genes in the fibrinogen-binding ability of CC17 strains.

## Results

### Prevalence of the *fbs* genes and of the *fbs* regulator genes in strains of CC17 and of the major other GBS clonal complexes

PCR was performed to characterize the presence of *fbs* genes (*fbsA* and *fbsB*) and their regulator genes (*rogB*, *rovS* and *rgf*) in a collection of 134 isolates representing the major clonal complexes of GBS species: CC1 (29 strains), CC10 (26 strains), CC17 (38 strains), CC19 (21 strains), and CC23 (20 strains) (Table 1). The *fbsA* gene was found in all CC17 and CC23 strains, in most strains of CC10 (92.3%) and CC1 (82.8%), and in only 23.8% of CC19 strains. The *fbsB* gene was found in all CC17 strains, in 75.0% of CC23 strains, and in only one CC19 strain (4.8%), whereas the CC1 and CC10 strains did not have an *fbsB* gene. The *rovS* gene was found in all strains of all the CCs. The *rogB* gene was found in all CC1, CC10, CC19 and CC23 strains and in only 21.0% of CC17 strains. The *rgf* locus was found in all CC17 and CC10 strains, in most CC1 strains (93.1%), and rarely in CC19 (14.3%) and CC23 (25.0%) strains. Thus, CC1 and CC10 strains shared the same profile containing the various *fbs* regulator genes and the *fbsA* gene, but not the *fbsB* gene. Most of CC19 strains lacked the *fbsA* and *fbsB* genes. The simultaneous presence of *fbsA* and *fbsB* genes was restricted to CC17 (100%) and CC23 (75%) strains. However, the regulator genes profile of these two groups of strains differed, since *rgf* locus and *rogB* gene were associated with CC17 and CC23, respectively. These data show that different *fbs* genes and *fbs* regulator genes profiles are related to the GBS phylogenetic lineages, and that CC17 strains have a unique configuration characterized by the *fbsA*, *fbsB*, *rovS,*and *rgf* genes combination and the absence of *rogB* gene.

**Table 1:** Prevalence of the *fbs* genes and of their regulator genes in a collection of 134 isolates belonging to the GBS major clonal complexes.

	Clonal complexes (CC)				
	CC17	CC19	CC23	CC1	CC10
	N° (%)	N° (%)	N° (%)	N° (%)	N° (%)
	n = 38	n = 21	n = 20	n = 29	n = 26
*fbsA*	38 (100.0)	5 (23.8)	20 (100.0)	24 (82.8)	24 (92.3)
*fbsB*	38 (100.0)	1(4.8)	15 (75.0)	0 (0.0)	0 (0.0)
*rovS*	38 (100.0)	21 (100.0)	20 (100.0)	29 (100.0)	26 (100.0)
*rogB*	8 (21.0)	21 (100.0)	20 (100.0)	29 (100.0)	26 (100.0)
*rgf*	38 (100.0)	3 (14.3)	5 (25.0)	27 (93.1)	26 (100.0)

### Expression of *fbsA* and *fbsB* genes in three non polar deletion *ΔrgfAC* mutant strains

**Figure 1:**
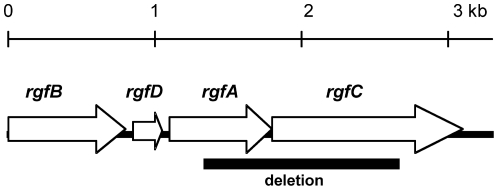
Schematic representation of the *rgf* locus. Open reading frames, direction of transcription and approximate gene sizes are indicated [25]. The segment that was deleted in *ΔrgfAC* mutant is represented as a heavy line below the genes.

**Figure 2:**
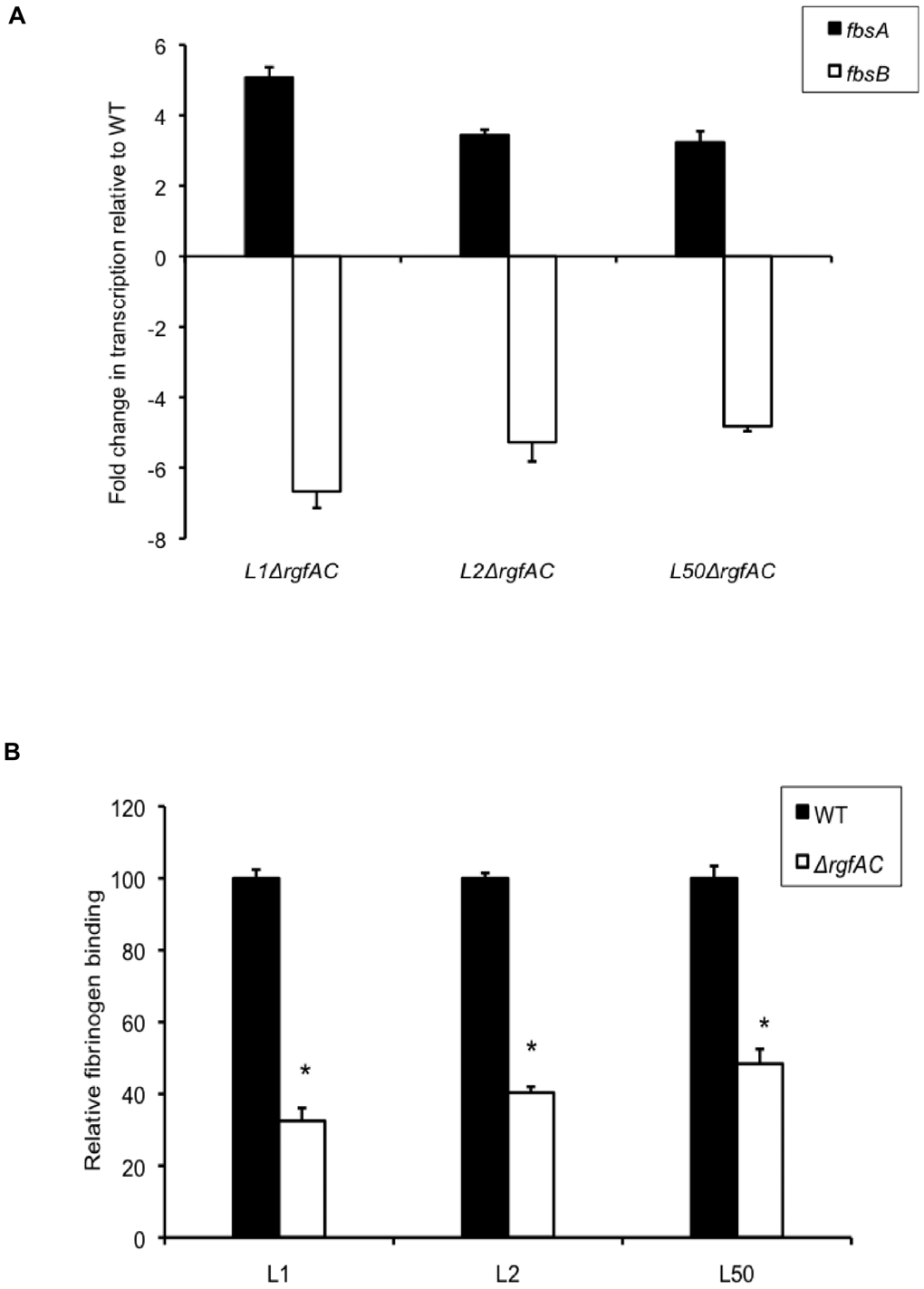
Properties of *ΔrgfAC* mutant strains. (A) Fold change in transcription levels of *fbsA* (filled boxes) and *fbsB* (open boxes) genes in the isogenic *ΔrgfAC* mutants as compared to the wild type L1, L2, and L50 strains (WT). The amount of transcripts of each gene was normalized to the amount of *gyrA* transcripts and expressed relative to the level of transcription in corresponding WT strain. Each experiment was performed at least three times. Boxes are means and bars are standard deviation of the means. (B) Binding ability to immobilized human fibrinogen of the isogenic *ΔrgfAC* mutants (open boxes) and the WT strains (filled boxes). Flat bottomed 96-well polystyrene plates were coated with 21 nM human fibrinogen and 5×10^6^ to 5×10^8^ CFU per ml were added for 90 min at 37°C. Binding ability was calculated from the ratio between the number of bound bacteria and the number of bacteria present in the inoculum. The level of fibrinogen binding of WT strains is arbitrarily reported as 100 and the fibrinogen-binding levels of the isogenic mutants are relative values. Each experiment was performed at least three times. Boxes are means and bars are standard deviation of the means. * indicates that the binding values of the mutant strains were significantly lower than the values of the corresponding WT strains, at a *P* value of <0.001.

As the *fbsA*, *fbsB* and *rgf* genes combination was strictly restricted to the CC17 strains and that the role of the RgfA/RgfC TCS on the *fbsB* and *fbsA* genes expression was not explored yet, we constructed non polar deletion *ΔrgfAC* mutants of serotype III strains belonging to CC17 phylogenetic lineage. Since phenotypes can be strain specific despite genetic similarity, we constructed mutants of three epidemiologically unrelated isolates, L1, L2 and L50 strains that were isolated from the cerebrospinal fluid (CSF) of neonates suffering from meningitis. In these mutants, the last 374 bp of *rgfA* gene encoding the DNA-binding domains, as well as the first 1,055 bp of the 1,278-bp rgfC gene were deleted (Fig. 1). By quantifying the transcription level of downstream gene, we checked that these mutations were non polar. Real time RT-PCR was then used to quantify the transcription levels of *fbsA* and *fbsB* genes in the three *ΔrgfAC* mutant strains and in the parental strains (Fig. 2A). As compared to L1, L2, and L50 wild type strains, the transcription levels of the *fbsB* gene were respectively 6.67±0.47-, 5.28±0.54-, and 4.82±0.14-fold decreased in *ΔrgfAC* mutants. By contrast, the transcription levels of the *fbsA* gene were respectively 5.07±0.30-, 3.45±0.05-, and 3.24±0.32-fold increased in *ΔrgfAC* mutant strains as compared to the wild type strains. These results indicate that RgfA/RgfC exerts a negative effect on the transcription of the *fbsA* gene, and that it activates the transcription of the *fbsB* gene in CC17 isolates.

To investigate whether RgfA/RgfC regulated *fbs* genes through the *rovS* gene that encodes an *fbsA* inhibitor, we quantified the *rovS* gene transcription levels in L1 *ΔrgfAC* mutant as compared to the parental strain, and found no significant difference (1.29±0.11-fold that of the wild type strain).

### Binding of the three *ΔrgfAC* mutant strains to immobilized human fibrinogen

**Figure 3:**
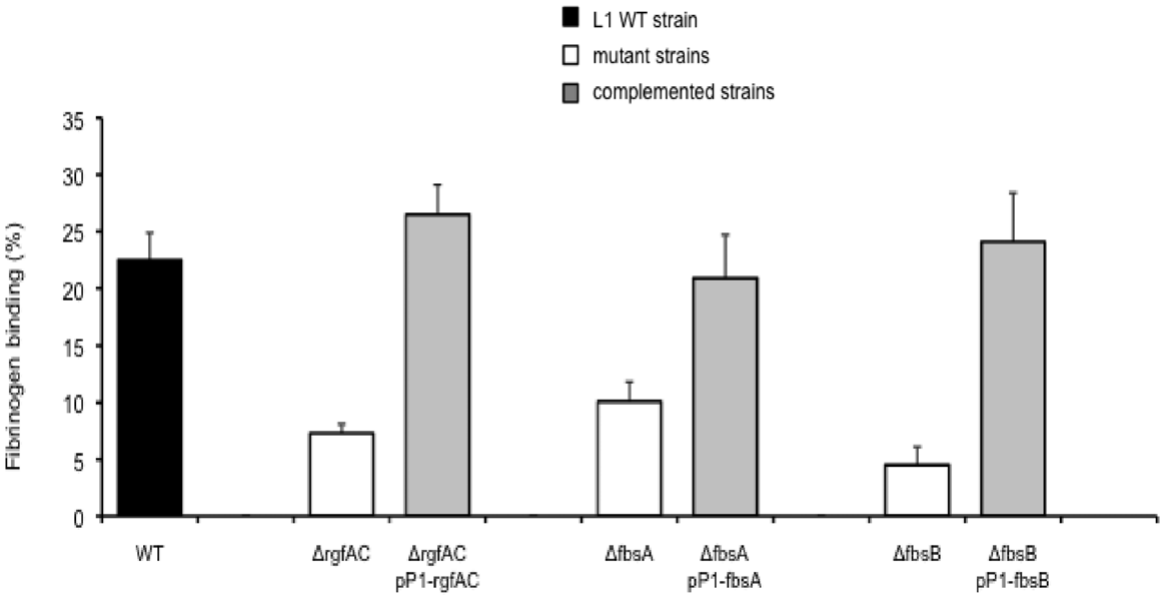
Binding ability to immobilized human fibrinogen of the wild type (WT) L1 strain, and of isogenic mutant and complemented strains for *rgfAC*, *fbsA*, and *fbsB* genes. Flat bottomed 96-well polystyrene plates were coated with 21 nM human fibrinogen and 5×10^6^ to 5×10^8^ CFU per ml were added for 90 min at 37°C. Binding ability was calculated from the ratio between the number of bound bacteria and the number of bacteria present in the inoculum. Each experiment was performed at least three times. Boxes are means and bars are standard deviation of the means. The binding values of the mutant strains were significantly lower, at a *P* value of <0.001, than the values of the L1WT strain and of the corresponding complemented strains carrying *rgfAC*, *fbsA*, and *fbsB* genes on the pP1 plasmid.

To further characterize the three *ΔrgfAC* mutant strains, we compared their fibrinogen-binding ability with that of the wild type L1, L2 and L50 strains. The percentage of bacteria that bound to fibrinogen was respectively 22.5% ±2.4%, 25.3% ±1.4%, and 23.2% ±3.4% for the three wild type strains, and 7.3% ±0.8%, 10.2% ±1.6%, and 11.2% ±1.1% for the isogenic *ΔrgfAC* mutants. Thus, as depicted in Fig. 2B, the three *ΔrgfAC* mutants showed respectively a 68%, 60%, and 52% decreased fibrinogen-binding ability as compared to L1, L2, and L50 wild type strains (*P*<0.001). Furthermore, plasmid-mediated expression of *rgfAC* in L1*ΔrgfAC* mutant strain restored its fibrinogen-binding ability to the wild-type level. Indeed, as shown in Fig. 3, the fibrinogen-binding ability of the complemented strain L1*ΔrgfAC*/pP1-*rgfAC* (26.5% ±2.6%) was significantly higher (*P*<0.001) than that of L1*ΔrgfAC* mutant (7.3% ±0.8%) and was similar to that of the wild type L1 strain (22.5% ±2.4%).

### Binding of *ΔfbsA*, *ΔfbsB,* and *ΔfbsAΔfbsB* mutant strains to immobilized human fibrinogen

**Figure 4:**
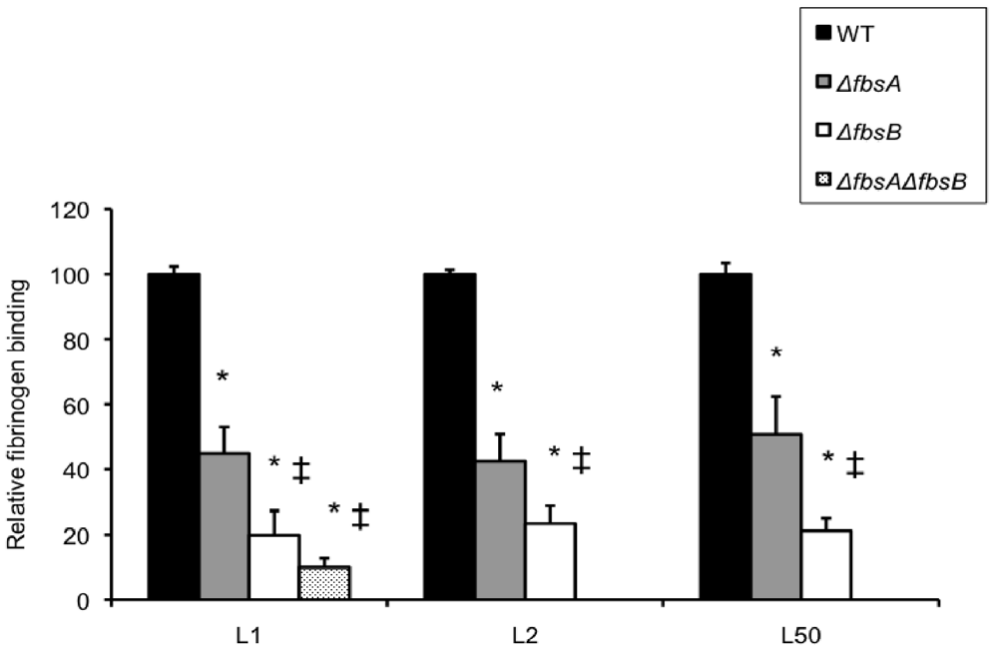
Binding ability to immobilized human fibrinogen of the wild type (WT) *S. agalactiae* strains and isogenic *ΔfbsA*, *ΔfbsB*, *ΔfbsAΔfbsB* deletion mutants. Flat bottomed 96-well polystyrene plates were coated with 21 nM human fibrinogen and 5×10^6^ to 5×10^8^ CFU per ml were added for 90 min at 37°C. Binding ability was calculated from the ratio between the number of bound bacteria and the number of bacteria present in the inoculum. The fibrinogen-binding level of WT L1, L2, and L50 strains is arbitrarily reported as 100 and the fibrinogen-binding levels of the various isogenic mutants are relative values. Each experiment was performed at least three times. Boxes are means and bars are standard deviation of the means. * indicates that the binding values of the mutant strains were significantly lower than the values of the corresponding WT strains, at a *P* value of <0.001. ‡ indicates that the binding values of the *ΔfbsB* and *ΔfbsAΔfbsB* mutant strains were significantly lower than the values of *ΔfbsA* mutant strains, at a *P* value of <0.001.

In order to investigate the relative contribution of FbsA and FbsB proteins in the interaction of GBS CC17 strains with human fibrinogen, *fbsA* and *fbsB* genes were deleted in the genome of L1, L2 and L50 strains, and both genes were deleted in the genome of L1 strain. The growth curves of the mutants and of the wild type parental strains in TH broth did not differ significantly. The wild type strains and their isogenic mutants (*ΔfbsA*, *ΔfbsB,* and *ΔfbsAΔfbsB*) were subsequently tested for their fibrinogen-binding ability. For L1 strain, the percentage of bacteria that bound to fibrinogen was 22.5% ±2.4% for wild type strain, 10.1% ±1.7% for *ΔfbsA*, 4.5% ±1.6% for *ΔfbsB*, and 2.2% ±0.7% for *ΔfbsAΔfbsB*. Thus, as shown in Fig. 4, deletion of *fbsA* gene reduced the fibrinogen-binding ability of L1 wild type strain by 55% (*P*<0.001), whereas deletion of *fbsB* gene resulted in a 80% decrease (*P*<0.001), and deletion of both *fbsA* and *fbsB* genes resulted in a 90% decrease of this ability (*P*<0.001). Similar results were obtained with *ΔfbsA* and *ΔfbsB* mutants of L2 and L50 strains (Fig. 4). Moreover, the fibrinogen-binding abilities of *ΔfbsB* and of *ΔfbsAΔfbsB* mutant strains were significantly lower than those of *ΔfbsA* mutant strains (*P*<0.001). In addition, plasmid-mediated expression of *fbsA* and of *fbsB* in L1*ΔfbsA* and in L1*ΔfbsB* mutants, respectively, restored their fibrinogen-binding ability to the wild-type level. Indeed, as shown in Fig. 3, the fibrinogen-binding ability of the complemented strains L1*ΔfbsA*/pP1-*fbsA* (20.9% ±3.8%) and L1*ΔfbsB*/pP1-*fbsB* (24.1% ±4.3%) were significantly higher (*P*<0.001) than those of L1*ΔfbsA* (10.1% ±1.7%) and L1*ΔfbsB* (4.5% ±1.6%) mutants and were similar to that of the wild type L1 strain (22.5% ±2.4%). Taken together, these data suggest a greater role of the fibrinogen-binding protein FbsB as compared to FbsA in the binding ability to human fibrinogen of CC17 GBS strains.

### Relative transcription level of *fbsA* and *fbsB* genes

**Figure 5:**
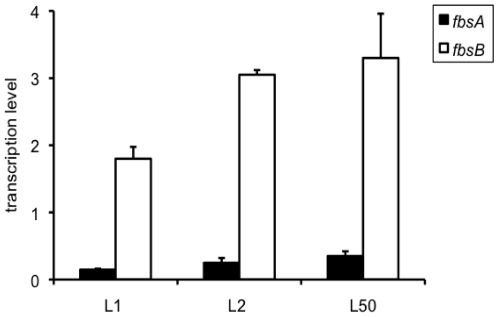
Transcription levels of *fbsA* and *fbsB* genes in three wild type CC17 strains. The amount of transcripts of *fbsA* gene (filled boxes) and *fbsB* gene (open boxes) in L1, L2, and L50 wild type strains was normalized to the amount of *gyrA* transcripts. Each experiment was performed at least three times. Boxes are means and bars are standard deviation of the means.

In order to determine if the greater role of FbsB as compared to FbsA in the binding ability to fibrinogen of CC17 strains was related to a higher transcription of *fbsB*, we quantified *fbsA* and *fbsB* gene transcripts by real-time PCR in the three wild type strains L1, L2 and L50. As shown in Fig. 5, the relative transcription level of *fbsB* gene was respectively 12.24±2.38-, 12.67±3.30- and 9.17±2.19-fold higher than that of *fbsA* gene for the three strains.

### Quantification of *fbsA* and *fbsB* gene transcripts in mutant strains

By real-time PCR, we quantified the transcript levels of *fbsA* and *fbsB* genes in *ΔfbsA*, *ΔfbsB,* and *ΔfbsAΔfbsB* mutants and in the parental strain L1. As expected, no *fbsA* transcripts were detected in *ΔfbsA* and in *ΔfbsAΔfbsB* mutants; likewise, no *fbsB* transcripts were detected in *ΔfbsB* and *ΔfbsAΔfbsB* mutants. Deletion of *fbsA* gene had no significant effect on the *fbsB* gene transcription, since in *ΔfbsA* mutant, the transcription level of *fbsB* was 1.4±0.2-fold that of the wild type strain. Similarly, deletion of *fbsB* gene had no significant effect on the *fbsA* gene transcription since the transcription level of *fbsA* in *ΔfbsB* mutant was 0.96±0.03-fold that of the wild type strain. These data demonstrate that the *fbsA* and *fbsB* genes expression are independent of each other.

## Discussion

Overrepresentation of CC17 clone among invasive neonatal strains is now well recognized worldwide [9]-[13], and highlights the fact that this clone is well adapted to neonate infection pathogenesis and may possess specific virulence traits that enhance CNS invasiveness in this population [14], [26]. We here studied the molecular events involved in the fibrinogen-binding ability of CC17 strains that were previously shown to bind significantly more strongly to human fibrinogen than strains of other lineages that constitute the species [17]. We first looked for the *fbs* genes and the *fbs* regulator genes in a collection of 134 GBS isolates belonging to the major GBS phylogenetic lineages. No gene was specific of either CC17 or other CCs strains, but specific gene combinations were related to particular CCs, indicating that fibrinogen binding is a multigenic process that results from various gene combinations. Only CC17 strains contained the *fbsA*, *fbsB*, and *rgf* genes combination. The *rogB* gene was rarely found in CC17 strains but present in all strains of other CCs. Accordingly, the *rogB* gene is missing in the sequenced genome of CC17 strain COH1 [27], and the absence of this gene was also reported in a collection of 20 CC17 strains [14]. Thus, each CC was characterized by a particular profile of *fbs* genes and *fbs* gene regulators that may account for differences in their fibrinogen-binding abilities.

As only CC17 strains contained the *fbsA*, *fbsB*, and *rgf* genes combination, we constructed non polar *ΔrgfAC* mutants of three serotype III CC17 strains that showed a 52% to 68% decreased binding ability to fibrinogen, a 4.8- to 6.7-fold decreased transcript level of *fbsB* gene, and at the same time a 3.2- to 5.1-fold increased transcript level of *fbsA* gene. These data demonstrate that RgfA/RgfC is implicated in *fbsB* gene activation and in *fbsA* gene inhibition. Spellerberg et al. have described that the response regulator RgfA and the histidine kinase RgfC display respectively 55% and 45% similarities with AgrA and AgrC of *Staphylococcus aureus* [25]. The accessory gene regulator (*agr*) locus consists of the four cotranscribed *agrBDCA* genes that regulate the expression of *S. aureus* virulence factors. In general, secreted proteins, including several of the known *S. aureus* toxins, are up-regulated by *agr* whereas surface proteins such as protein A and extracellular matrix adhesins are down-regulated [28]–[32]. Thus, our results showing the increased transcription of the *fbsA* gene encoding a surface protein, as well as the decreased transcription of the *fbsB* gene encoding a secreted protein in *ΔrgfAC* mutant strain, are in agreement with the control of cell surface and secreted molecules through *agr* locus of *S. aureus*. Similarly, Spellerberg et al. found that *rgf* locus down regulated the surface-anchored C5a peptidase *scpB* gene [25]. Response regulators may modify genes expression by direct binding to the genes promoters or by action on other regulators that, in turn regulate target genes expression [16], [33]–[36]. As RovS was shown to directly bind to the promoter of *fbsA* and hence to negatively regulate its transcription [24], we quantified *rovS* transcript levels in a *ΔrgfAC* mutant in order to determine whether RgfA/RgfC regulated *fbsA* through *rovS* gene. We found that the *rovS* transcription was not altered in this mutant. Thus, it is likely that RgfA/RgfC- and RovS- mediated control of *fbsA* are independent of each other. Finally, RgfA/RgfC appears to be an important multigene regulator in the hyper virulent CC17 lineage, and is therefore worthy of further disease association studies.

Phenotypic comparison of three CC17 wild type strains with their mutants deleted for the *fbsA* and *fbsB* genes demonstrated that FbsB protein was the major fibrinogen binding protein of these strains. Indeed, inactivation of the *fbsB* gene substantially reduced fibrinogen-binding ability (78 to 80%), while deletion of *fbsA* gene reduced only partially (49 to 57%) this ability. Our findings showing the implication of both FbsA and FbsB proteins in the fibrinogen-binding ability of CC17 strains with a major role of FbsB are in contrast with previous reports suggesting that FbsA protein was the major fibrinogen-binding protein in the CC23 serotype III 6313 GBS strain [18], [20], [21]. The reasons for these phenotype differences between isolates are not completely understood but can be related to differences in *fbs* genes regulation depending on the genetic background of the GBS strains. Interestingly, CC17 strains and CC23 6313 strain that both contain *fbsA* and *fbsB* genes, possess a distinct *fbs* regulator profile: contrarily to CC17 strains, 6313 strain has no functional *rgfBDAC* locus and moreover possesses *rogB* gene [23]. This configuration that leads to an up-regulation of the *fbsA* gene, is thus in agreement with the major role of FbsA in the fibrinogen-binding process of CC23 strains. On the contrary, the higher implication of FbsB as compared to FbsA in the fibrinogen-binding ability of CC17 strains may be explained by the fact that the *fbsA* gene expression is inhibited by two mechanisms: on one hand by the RgfA/RgfC inhibitor, and on the other hand by the absence of the RogB activator. Indeed, in the three CC17 strains studied, the mean transcription level of *fbsB* gene was significantly higher (11.4±1.9-fold) than that of *fbsA* gene. Thus, depending on the genetic background of the GBS strains, several *fbs* regulatory circuits may be defined and may account for the differences in the relative implication of *fbs* genes in GBS binding-ability to human fibrinogen.

In conclusion, our results demonstrate that a specific combination of *fbs* genes and *fbs* regulator genes may account for the CC17 strains enhanced ability to bind to human fibrinogen, a host protein whose synthesis is dramatically increased during inflammation or under exposure to stress such systemic infections [37].

## Materials and Methods

### Bacterial strains and growth conditions

A total of 134 unrelated human strains representing the genetic diversity of *S. agalactiae* species were included in the present study: 52 strains from the CSF of infected neonates, 16 strains from the gastric fluid of colonized asymptomatic neonates, 26 strains from vaginal swabs of colonized asymptomatic pregnant women, and 40 strains from infected nonpregnant adult patients. Neonatal specimens were obtained from infected neonates suffering of meningitis and from non infected neonates with risk factor, and vaginal specimens were obtained from asymptomatic pregnant women during the process of routine clinical diagnostic procedures, as part of the usual prenatal and postnatal screening. All these strains were isolated between 1986 and 1990 from 25 general hospitals throughout France [7]. According to the information we obtained from the Institutional Review Board (more than 20 years ago), this type of strains did not require an ethics approval and the patient consent. Adult specimens were isolated from sites of infection (skin, osteoarticular, and blood infections) of patients admitted to hospitals in various regions of France from 2002 to 2008. The local ethical committee (CPP, Comité de Protection des Personnes, Tours-Centre) exempted the study of adult specimens from review because they were of existing diagnostic specimens, and waived the need for consent due to the fact that the samples received were analyzed anonymously.

All strains had previously been serotyped on the basis of capsular polysaccharides or by PCR [7], [38]. Six serotypes were identified i.e. serotypes Ia (19 strains), Ib (18 strains), II (17 strains), III (51 strains), IV (2 strains), and V (23 strains), and four strains were not typeable. All strains had previously been analyzed by MLST according to Jones et al. [5]. Strains were grouped into clonal complexes (CCs) that include isolates sharing five to seven identical alleles.

GBS strains were stored at −80°C in Schaedler-vitamin K_3_ broth (bioMérieux, Marcy l'Etoile, France) with 10% glycerol. The bacteria were grown for 24 h on 5% horse blood Trypticase soja (TS) agar plates (bioMérieux) at 37°C.

*Escherichia coli* DH5α and MC1061 were used for cloning purposes. They were grown at 37°C in Luria Broth. *E. coli* and GBS clones carrying the pG^+^host5 and pP1 plasmids were selected in the presence of 300 µg/ml and 2 µg/ml erythromycin, respectively.

### Construction of *S. agalactiae* mutants

The thermosensitive plasmid pG^+^host5 [39] was used for the construction of mutants of the CC17 serotype III L1 wild type GBS strain deleted for *fbsA* gene, for *fbsB* gene, for both genes, and for *rgfAC* genes by a method previously described by Schubert et al. [20]. For the deletion of *fbsA* gene, two DNA fragments flanking the *fbsA* gene were amplified by PCR using the primer set fbsA_del1 (5′-CCGCGGATCCGAATATGCTACCATCAC)/fbsA_del2 (5′-*CCCATCCACTAAACTTAAACA* TTCCTGATTTCCAAGTTC) and the primer set fbsA_del3 (5′-*TGTTTAAGTTTAGTGGATG GG*GCTGCGGTTTGAGACGC)/fbsA_del4 (5′-TGGCACAAGCTTTACCTGCTGAGCGAC TTG). Complementary DNA sequences in the primers fbsA_del2 and fbsA_del3 are shown in italics, and the *Bam*HI and *Hind*III restrictions sites in primers fbsA _del1 and fbsA _del4 are underlined. The *fbsA*-flanking PCR products were mixed in equal amounts and subjected to a crossover PCR with the primers fbsA _del1 and fbsA _del4, resulting in one PCR product that carried the two *fbsA*-flanking regions. The crossover PCR product and the plasmid pG^+^host5 were digested with *Bam*HI and *Hind*III, ligated and transformed into *E. coli* DH5α. The resulting plasmid, pG^+^host5*ΔfbsA*, was electroporated into GBS L1 isolate [40], and transformants were selected by growth on erythromycin (2 µg/ml) TS agar at 28°C. Cells in which pG^+^host5*ΔfbsA* had integrated into the chromosome were selected by growth of the transformants at ≥37°C with erythromycin selection. Four of such clones were serially passaged for 6 days in Todd-Hewitt (TH) broth (Sigma, St Quentin Fallavier, France) at 28°C without antibiotic pressure to facilitate the excision of plasmid pG^+^host5*ΔfbsA*, leaving the desired *fbsA* deletion in the chromosome. Dilutions of the serially passaged cultures were plated onto TS agar and single colonies were tested for erythromycin susceptibility to identify pG^+^host5*ΔfbsA* excisants.

The *fbsB* gene was deleted in the chromosome of L1 wild type strain and in L1*ΔfbsA* mutant strain as described above, using the following primers: fbsB_del1 (5′-*CCGCGGATCCGTCATGTTACTAATCTTATGC*), *fbsB*_del2 (5′-*CCCATCCACTAAACTTA ACA*CAATCCAAAACGCAATAGG), fbsB_del3 (5′-*TGTTTAAGTTTAGTGGATGGG*GATC AAGCTTTTGTAGCTAG), and fbsB_del4 (5′-GGGGGTACCCTTCATTAACAATATCTG AG). The non polar deletion mutant strain *ΔrgfAC* was constructed by deletion of the last 374 bp of the *rgfA* gene encoding the DNA-binding domain, and of the first 1.055 bp of the *rgfC* gene in the chromosome of L1 wild type strain (Fig. 1), as described above using the following primers: rgfA_del1 (5′-CCGCGGATCCTCAACAGGCACGTTTAGAGAGA), rgfA_del2 (5′-*CCCATCCACTAAACTTAAACA*AACGTCTTCAATCCTTCTGCT), rgfC_del3 ( *5′*-*TGTTTAAGTTTAGTGGATGGG*GATAACGCTATTGAGGCATCT), and rgfC_del4 (5′-GGGGGTACCATCACTGGTGGTGGTTGGAT). Complementary DNA sequences in the primers fbsB_del2 and fbsB_del3 and in the primers rgfA_del2 and rgfC_del3 are shown in italics, and the *Bam*HI and *Kpn*I restrictions sites in primers fbsB _del1 and fbsB_del4 and in primers rgfA _del1 and rgfC_del4 are underlined.

Successful gene deletions in *ΔfbsA*, *ΔfbsB*, *ΔfbsAΔfbsB*, and *ΔrgfAC* mutant strains were confirmed by PCR using primers flanking the deletion site and then by sequencing the amplified fragment.

### Plasmid-mediated expression of *fbsB*, *fbsA*, and *rgfAC* in *S. agalactiae*

The pP1 plasmid [41] was used for complementation analysis of L1 *ΔfbsB*, *ΔfbsA* and *ΔrgfAC* isogenic mutants. The *fbsA*, *fbsB,* and *rgfAC* genes, including their ribosomal binding sites, were amplified from chromosomal DNA of L1 GBS strain by PCR using the Herculase Hotstart DNA Polymerase (Stratagene, Santa Clara, USA) and the following primer sets: fbsB_compl1 (5′-GGGGAGCT CTATTATCTCGTGATAAGTTTTTGATG)/fbsB_compl2 (5′-CCGCGGATCCTTTAAGAT CGCCTTGATAGCAG) for *fbsB* gene, fbsA_compl1 (5′-GGGGAGCTCAAAAGTAAGGAG AAAATTAATTGTTC)/fbsA_compl2 (5′-CCGCGGATCCCCGATTCCTTTTTATTGATTG C) for *fbsA* gene, and rgf_compl1 (5′-GGGGAGCTCTCAACAGGCACGTTTAGAGAG)/rgf_compl2 (5′-CCGCGGATCCATCACTGGTGGTGGTTGGATTG) for *rgfAC* genes. The *Sac*I *and Bam*HI restriction sites used for cloning are underlined. The *fbsB*, *fbsA* and *rgfAC*-containing PCR products and the plasmid pP1 were digested with *Sac*I and *Bam*HI, ligated and transformed into *E. coli* MC1061. The plasmid pP1 and the resulting plasmids pP1-*fbsB*, pP1-*fbsA* and pP1-*rgfAC* were subsequently transformed by electroporation into the corresponding L1 isogenic mutants *ΔfbsB*, *ΔfbsA* and *ΔrgfAC*.

### DNA amplification

Bacterial genomic DNA (20 ng), extracted and purified by conventional methods [42], was used as the template for PCR assays. All primers used (Table 2) were purchased from Eurogentec (Seraing, Belgium). The widely distributed *lmb* gene [43], used as a control, was amplified with primer set *lmb*130/*lmb*970. Four primer sets designed in various sites of the *rogB* gene were used to amplify the *rogB* gene, and two primer sets were used to amplify *rgfBDAC* locus, *rovS*, *fbsA*, and *fbsB* genes. The mixture (20 µL) contained primers (0.2 µM each), deoxynucleoside triphosphates (200 µM each), *Taq* DNA polymerase (0.5 U) (Roche Diagnostics, Mannheim, Germany), and 1.5 mM MgCl_2_, in 1X buffer. The PCR consisted of an initial 5 min hold at 94°C followed by 30 cycles, each of 1 min denaturation at 94°C, 0.5 min annealing at 55°C or at 50°C, and 1 min elongation at 72°C, followed by a final 10 min elongation step at 72°C (GeneAmp® PCR System 2700, Applied Biosystems, Foster City, USA).

**Table 2:** Oligonucleotide primers.

Name of primer	Nucleotide sequence (5′-3′)	Amplified fragment size	Target genes in reference strain	GBS reference strain
*lmb130*	GTTGTGAGTTTAGTAATGATAGC	840 bp	*lmb*	R268 [43]
*lmb*970	GATATGTCTTGTTCCGCTTG			
*rogB*59	GCTATGATTACTACCCTTCCATTACTC	130 bp	*rogB*	6313 [23]
*rogB*189	TTCGATATTCAGAGAGAGTTGACTG			
*rogB*298	GATTCAGGCAGGTTCCCTTT	891 bp		
*rogB*1189	CGGCTATTTGTATCGGAGGA			
*rogB*350	GTGCAACTGCTTATCGCATAC	794 bp		
*rogB*1144	GGTGAGCACAAAGGAGAAGAA			
*rogB*822	TTGGTCTGAGAAGCGTATCG	131 bp		
*rogB*953	GCAACTTTTACCAACTCGTCA			
*rgfB1*	TCTATGGCAAAATGCTTAACG	1086	*rgfBD*	O90R [25]
*rgfD155*	TCTCTAAACGTGCCTGTTGAA			
*rgfD89*	ACGAGGAGACGAAAGTGAAT	2277	*rgfDAC*	
*rgfC*	CGCAAAGTTCTATGGTTCAAAA			
*rovS114*	CAAGGTTTGAGAGAGGAGAGTCA	631 bp	*rovS*	NEM 316 [24]
*rovS745*	TCCTGAAGAAGTATCACCAAGTTTT			
*rovS82*	AGCAGATGAGCACCTATCCA	146 bp		
*rovS228*	TGAGTGTGCGCCTTAGAATG			
*fbsA282*	CAACTTATAGGGAAAAATCCAC	123 bp	*fbsA*	176H4A [20]
*fbsA 405*	AGTTAACATCGGTCTATTAGC			
*fbsA*86	ATCAAGTCCTGTATCTGCTAT	469 bp		
*fbsA*555rc	TTCATTGCGTCTCAAACCG			
*fbsB354*	GCGATTGTGAATAGAATGAGTG	129 bp	*fbsB*	NEM316 [18], [45]
*fbsB483*	ACAGAAGCGGCGATTTCATT			
*fbsB*143	TCGGTCATAAAATAGCGTATGG	1567 bp		
*fbsB*1710rc	AAGAATTCAACGGTCGGCTTCGT			
*gyrA*	CGGGACACGTACAGGCTACT	128 bp	*gyrA*	NEM316 [45]
*rgyrA*	CGATACGAGAAGCTCCCACA			

### Quantification of specific transcripts with real-time PCR

*S. agalactiae* L1 wild type isolate and isogenic mutant strains were grown in 50 ml of TH broth to stationary growth phase ([OD_595_]  = 1.2). Bacterial cells pelleted by centrifugation were lysed mechanically with 0.25–0.5 mm glass beads (Sigma) in Tissue Lyser (Qiagen, Hilden, Germany) for 6 min at 30 Hz. RNA was purified by using the RNeasy Mini kit (Qiagen), then treated with DNase using DNAfree kit (Ambion, Cambridgeshire, UK) and checked for DNA contamination by PCR amplification without prior reverse transcription. As no amplicons were obtained, the possibility of DNA contamination during RNA preparation could be excluded. Reverse transcription of 1 µg of RNA was performed with random hexanucleotides and the Quantiscript Reverse Transcription kit (Qiagen). Real-time quantitative PCR was performed in a 25 µl reaction volume containing cDNA (50 ng), 12.5 µl QuantiTect SYBR Green PCR Master Mix (Qiagen), and 0.3 µM of each gene-specific primer set in an iCycler iQ detection system (BioRad). The specific primers for *gyrA*, *fbsA* (*fbsA282*/*fbsA405*), *fbsB* (*fbsB354*/*fbsB483*) and *rovS* (*rovS82*/*rovS228*) genes were used (Table 2). The PCR consisted of an initial 15 min hold at 95°C followed by 40 cycles, each of 15 sec at 94°C, 30 sec at 58°C, and 30 sec with fluorescence acquisition at 72°C. The specificity of the amplified product was verified by generating a melting-curve with a final step of 50 cycles of 10 sec at an initial temperature of 70°C, increasing 0.5°C each cycle up to 95°C. The quantity of cDNA for the investigated genes was normalized to the quantity of *gyrA* cDNA in each sample. The *gyrA* gene was chosen as an internal standard since gyrase genes represent ubiquitously expressed house-keeping genes that are frequently used for the normalization of gene expression in quantitative reverse transcription-PCR experiments [23], [24], [44]. The transcription levels of *fbsB, fbsA* and *rovS* genes in wild type isolates were treated as the basal levels. Each experiment was performed at least three times. Gene transcript levels of isogenic mutant strains were expressed as fold-transcript levels relative to those of the parental strains. A twofold difference was interpreted as a significant difference in expression between the parental and the mutant strains.

### Binding of *S. agalactiae* to immobilized human fibrinogen

All binding assays were performed in triplicate as previously described [17]. Briefly, flat bottomed 96-well polystyrene plates were coated for 18 h at 4°C with 21 nM human fibrinogen (Diagnostica Stago, Asnières, France) diluted in phosphate-buffered saline (PBS) (150 mM NaCl, 10 mM sodium phosphate, pH 7.2). Bacterial cells were harvested from overnight cultures in TH broth and resuspended in PBS. Fibrinogen-coated wells were washed, and then 50 µl of PBS containing 5×10^6^ to 5×10^8^ CFU per ml were added to each well. After incubation for 90 min at 37°C, non binding bacteria were removed by washing with PBS. Bound bacteria were subsequently unbound by the addition of a 0.01% solution of protease/serine protease mix (Sigma) to each well, then the viable bacteria were quantified by plating serial dilutions onto TS agar plates. The percentage of binding to human fibrinogen was obtained by the ratio between the number of bound bacteria and the number of bacteria present in the inoculum. Statistically significant difference in fibrinogen-binding ability was determined at 95% confidence level (*P*<0.05) for a two-sample t-test assuming unequal variance.
